# Efficacy of therapist-supported online remote behavioral therapy for tic disorders: a systematic review and meta-analysis

**DOI:** 10.3389/fpsyt.2025.1521947

**Published:** 2025-06-13

**Authors:** Xiaolei Xu, Kangsheng Zhu, Weiyi Wang, Tianyu Zhao, Congrui Fu

**Affiliations:** ^1^ School of Nursing, Hebei Medical University, Hebei, Shijiazhuang, China; ^2^ Department of Anesthesiology, The Forth Hospital of Hebei Medical University, Shijiazhuang, China; ^3^ Department of Immunology and Pathobiology, Hebei University of Chinese Medicine, Shijiazhuang, China

**Keywords:** tic disorders, tics, behavior therapy, internet-delivered, online

## Abstract

**Introduction:**

Recently, several studies about therapist-supported online remote behavioral therapy (TSORBT) have been showed effective for tic disorders (TD). With the increasing adoption of telemedicine, a systematic review of existing evidence is crucial to confirm the efficacy of TSORBT in treating TD.

**Aim:**

We aim to assess the efficacy of TSORBT on the treatment of TD.

**Method:**

This review followed PRISMA guidelines. We searched PubMed, Web of Science, Cochrane, and Embase up to September 2024 for clinical trials on TSORBT’s efficacy in TD. Two researchers independently screened studies, focusing on the Total Tic Severity Score of the Yale Global Tic Severity Scale (YGTSS-TTSS) and other subscores of the YGTSS-motor tic score (YGTSS-MTS), YGTSS-vocal tic score (YGTSS -VTS), YGTSS-impairment score and Parent Tic Questionnaire (PTQ). Risk of bias was assessed using Cochrane RoB 2. Data were analyzed in RevMan 5.4, with outcomes expressed as mean difference (MD) and 95% CI.

**Results:**

In total, 2764 articles were identified for screening. Nine studies involving 1049 participants, with 527 assigned to the TSORBT and 522 to the control were included. TSORBT exhibited potential in addressing YGTSS-TTSS (MD = -2.22, 95% CI: [-3.16, -1.29], *P*<0.00001), YGTSS-MTS (MD = -2.17, 95% CI: [-3.39, -0.96], *P*=0.0004), YGTSS-impairment score (MD= -1.69, 95% CI: [-3.26, -0.12], *P*=0.03) and PTQ score (MD= -6.05, 95% CI: [-8.65, -3.44], *P*<0.00001);. Subgroup analyses revealed that TSORBT demonstrated nearly the same efficacy as face-to-face BT in addressing YGTSS-TTSS (MD = -0.38, 95% CI: [-3.20, 2.43], *P*=0.79), but more effective than online psychoeducation (MD = -2.37, 95% CI: [-3.64, -1.10], *P* = 0.0002).

**Conclusion:**

The current study demonstrates the promising therapeutic efficacy of TSORBT in TD. Further rigorously designed studies, particularly those assessing long-term treatment durability, are warranted to consolidate the evidence base.

## 1 Introduction

Tic disorders (TD) is a prevalent neurodevelopmental disorder, which affected up to 1% of children and adolescents, distinguished by abrupt and repetitive movements or vocalizations, and in most patients, symptoms persist into adulthood (1, 2). These disorders can be categorized into three types based on the duration of the condition, with a one-year threshold, as well as the clinical features: transient TD, chronic motor or vocal TD, and Tourette Syndrome (TS) (3). In addition, many TD children have behavioral or psychological disorders, such as attention deficit hyperactivity disorder, obsessive-compulsive disorder, and learning difficulties, which have adverse effects on children’s learning, life, social life, and psychology (4–6).

Currently, the predominant approach to managing TD involves pharmaceutical interventions (7, 8). Nonetheless, the utilization of these medications is accompanied by notable drawbacks, including the potential for severe and diverse adverse effects, as well as limitations in their long-term applicability (9, 10). In recent decades, behavior therapy (BT) has emerged as a successful modality for effectively addressing TD (11–14). Both Habit Reversal Training (HRT) and Comprehensive Behavioral Intervention (CBIT), as well as Exposure and Response Prevention (ERP), are recommended as primary interventions for TD by European clinical guidelines (15). Despite this clear recommendation, BT is rarely available in most countries due to barriers, such as lack of trained therapists and personal and domestic considerations concerning time, distance and costs (16, 17). During the past 10 years, the development of online remote BT has progressed significantly in treating various mental health conditions, including anxiety disorders, depressive symptoms and severe health anxiety (18–20). Recently, several studies have shown that therapist-supported online remote BT (TSORBT) is efficacious in treating TD (21–25). The potential benefits of TSORBT are customizability, time-effectiveness, geographic flexibility, consistency and availability (26).

While these findings are promising, the studies were conducted in different countries, with different interventions, and even yielded some contradictory results. In addition, with the increasing adoption of telemedicine, a systematic review of existing evidence is crucial to confirm the efficacy of TSORBT in treating TD. Specifically, the aims of the present study were to: (1) evaluate the TSORBT’s effects on tic severity in patients with TD. (2) assess the therapeutic efficacy of TSORBT in comparison with traditional face-to-face (f2f) therapy and online psychoeducational programs. This review not only informs clinical decision-making regarding TSORBT implementation in TD populations but also provides guidance for future research directions to optimize therapeutic protocols. Furthermore, it underscores the importance of consolidating this knowledge in the context of the growing applications of telemedicine.

## 2 Methods

### 2.1 Study design

The present systematic review and meta-analysis followed the Preferred Reporting Items for Systematic Reviews and Meta-analyses (PRISMA) 2020 statement (27), encompassing the published clinical trials on using TSORBT for TD (**Supplementary S1**: PRISMA Checklist). This trail was registered in PROSPERO (CRD42023432189).

### 2.2 Literature search

We searched the PubMed, Web of Science, Cochrane and Embase using extensive search strategies. The literature was searched from inception to September 2024. We included clinical trials investigating the efficacy of TSORBT for TD. We constructed a thorough search string using the entry terms of relevant keywords (“Tic Disorders”, “Tourettes Syndrome”, “Tics”, “Internet-Delivered”, “online remote”, “Internet”, “web”, “Videoconference” and “behavior therapy”). The search was restricted to human studies and the language of publications was restricted to English. Detailed search strategies can be seen in **Supplementary S2**.

### 2.3 Eligibility criteria: types of studies, participants and intervention

We selected eligible studies based on pre-identified criteria. We included only clinical trials, and our PICOS (Population, Intervention, Comparison, Outcome) was as follows:

Population: patients diagnosed with TD or TS
Intervention: treatment with TSORBT
Comparison: face to face BT or psychoeducation.
Outcome: tic severity were measured by the Total Tic Severity Score of the Yale Global Tic Severity Scale (YGTSS-TTSS) and other subscores of the YGTSS-motor tic score (YGTSS-MTS), YGTSS-vocal tic score (YGTSS -VTS), and YGTSS-impairment score. We also focus on Parent Tic Questionnaire (PTQ).

This meta-analysis included studies that met the following criteria: Patients: (1) Participants had to have a clinical diagnosis of TD based on the Diagnostic and Statistical Manual of Mental Disorders, 4^th^ ed. (DSM-IV). (2) Diagnostic and Statistical Manual of Mental Disorders, 5th ed. (DSM-5). (3) The International Classification of Diseases, Tenth Revision; Exclusion criteria included (1) Receiving acupuncture or massage therapy; (2) open-label studies. (3) review articles, conference abstracts, editorials, commentaries, case reports.

### 2.4 Screening of the studies

The process of literature screening involves two researchers independently assessing the inclusion and exclusion criteria for the literature. In cases where there are discrepancies in the inclusion of literature, a third party is consulted to make the final decision. Subsequently, the literature data is extracted, encompassing various elements such as title, author’s name, publication year, randomization method, sample sizes of the study and control groups, intervention method, and outcome evaluation indices. If data is missing in the paper, we contact the author directly to obtain it.

### 2.5 Assessment of risk of bias

We have used the Cochrane risk-of-bias 2 tool (RoB 2) for randomized controlled trials (RCTs) (28). For each study, two authors independently assessed the risk of bias, and a third author resolved any differences.

### 2.6 Statistical analysis

Data were analyzed using RevMan 5.4 software provided by the Cochrane Collaboration (Cochrane Collaboration, Copenhagen, Denmark). Continuous data was presented as the mean difference between groups with a 95% confidence interval (CI). A p value of ≤ 0.05 was deemed statistically significant. The heterogeneity in the data was examined through I-square and p value for significance. The Cochrane Handbook’s guidelines for meta-analysis were followed when interpreting the I-square test (low heterogeneity: I^2^ = ≤ 25%; moderate: I^2^ = 25 to ≤ 50%; high: I^2^ = > 75%) and a p value < 0.05. Visual inspection of a funnel plot and Egger’s test were performed to assess publication bias (Stata 17.0). We also conducted subgroup analyses based on the different control. TSORBT have shown great efficacy, but when compared with the mean of face to face or online psychoeducation, which one showed better efficacy need to be clarified. Thus, subgroup analysis was performed to identify the differences between them.

## 3 Results

### 3.1 Results of the search

**Figure 1:**
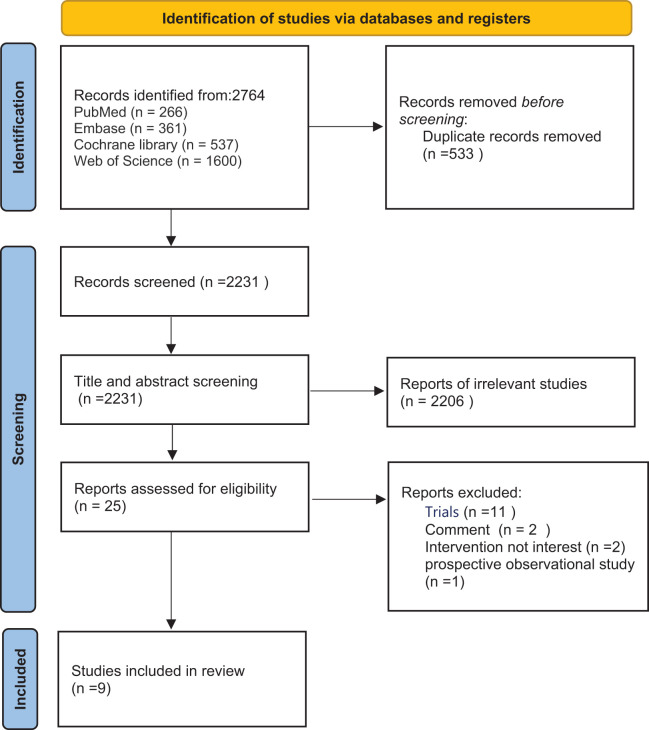
Flow chart of the included studies.

A total of 2764 studies were searched in the target databases. Among these, 533 duplicates were eliminated, 2231 were assessed through titles and abstracts, and 25 were evaluated by reading full-texts. Ultimately, nine studies were subjected to analysis (the PRISMA flow diagram in **Figure 1**).

### 3.2 Study characteristics and quality assessment

**Table 1** presents a comprehensive overview of the characteristics of these studies. Notably, only one RCT focused on adults, while the remaining studies encompassed children and adolescents. The nine studies involved 1049 participants and 84.2% were male, with 527 assigned to the TSORBT and 522 to the control. The treatment groups that were considered were TSORBT of various online remote BT. All the study followed up more than 2 months.

**Table 1:** Characteristics of included studies.

Study and year	Study design	Comparison Condition	Total sample size	Age (Mean, SD)	Sex, Male (%)	Follow up	Comorbidities	Main findings
Prato 2022 (25)	RCT	Online BT vs face-to-face BT	40	13.5 (2.0)	90%	2 month	+OCD 24 +LD 17 +Anxiety 17	Online remote and f2f BT are equally effective in the treatment of tics
Himle 2012 (29)	Randomized pilot trial	videoconference BT vs face-to-face BT	18	11.6 (2.7)	94%	4 month	+ADHD 28% +OCD 22% +Anxiety 33%	videoconference is a viable option for disseminating Behavioral Intervention for Tics
Ricketts 2016 (30)	Randomized pilot trial	Voice over Internet vs waitlist	20	12.3 (2.39)vs 11.9 (2.41)	65%	10 weeks	+ADHD 20% +OCD 5% +Anxiety 5%	Significantly greater reductions in tic severity were found in CBIT-VoIP relative to waitlist.
Rachamim 2022 (22)	RCT	ICBIT vs waitlist	41	11.26 (1.94)	70.73%	6 month	+ADHD43.9% +OCD 31.7% +Anxiety 39.0%	ICBIT offers an acceptable and safe alternative for TD.
Haas 2022 (24)	RCT	ICBIT vs placebo and face-to-face CBIT.	161	35.66 (12.4)	69.6%	6 month	NA	ICBIT is effective in the treatment of tics and results in greater self-responsibility once accepted by the patient
Hollis 2021 (31)	RCT	Online therapist-supported ERP vs psychoeducation	224	12.4 (2.1) vs 12.2 (2.0)	79%	3 month	+ADHD22.5% +OCD 5% +Anxiety 27.2%	Online therapist-supported ERP is an effective behavioral therapy for reduction in tic symptoms which has the potential to greatly increase the availability of effective behavioral treatment for children and adolescents with tic disorders.
Andrén 2022 (32)	RCT	Therapist-supported internet delivered ERP vs psychoeducation	221	12.1 (2.3)	68.8%	3 month	+ADHD15.4% +OCD 7.7% +Anxiety 14%	Therapist-supported internet-delivered ERP and education were both associated with significantly and clinically meaningful improvements in tic severity.
Hollis 2023 (23)	RCT	Online therapist-supported ERP vs psychoeducation	182	12.4 (2.1) vs 12.2 (2.0)	79%	18 month	+ADHD22.5% +OCD 5% +Anxiety 27.2%	Remotely delivered online ERP is a clinical and cost effective intervention with durable benefits extending for up to 18 months.
Andrén 2024 (33)	RCT	Therapist-supported internet delivered ERP vs psychoeducation	208	12.1 (2.3)	68.8%	12 month	+ADHD15.4% +OCD 7.7% +Anxiety 14%	No statistically significant changes in tic severity from the 3-month to the 12-month follow-up for either the internet-delivered ERP group or the internet-delivered psychoeducation group.

**Figure 2:**
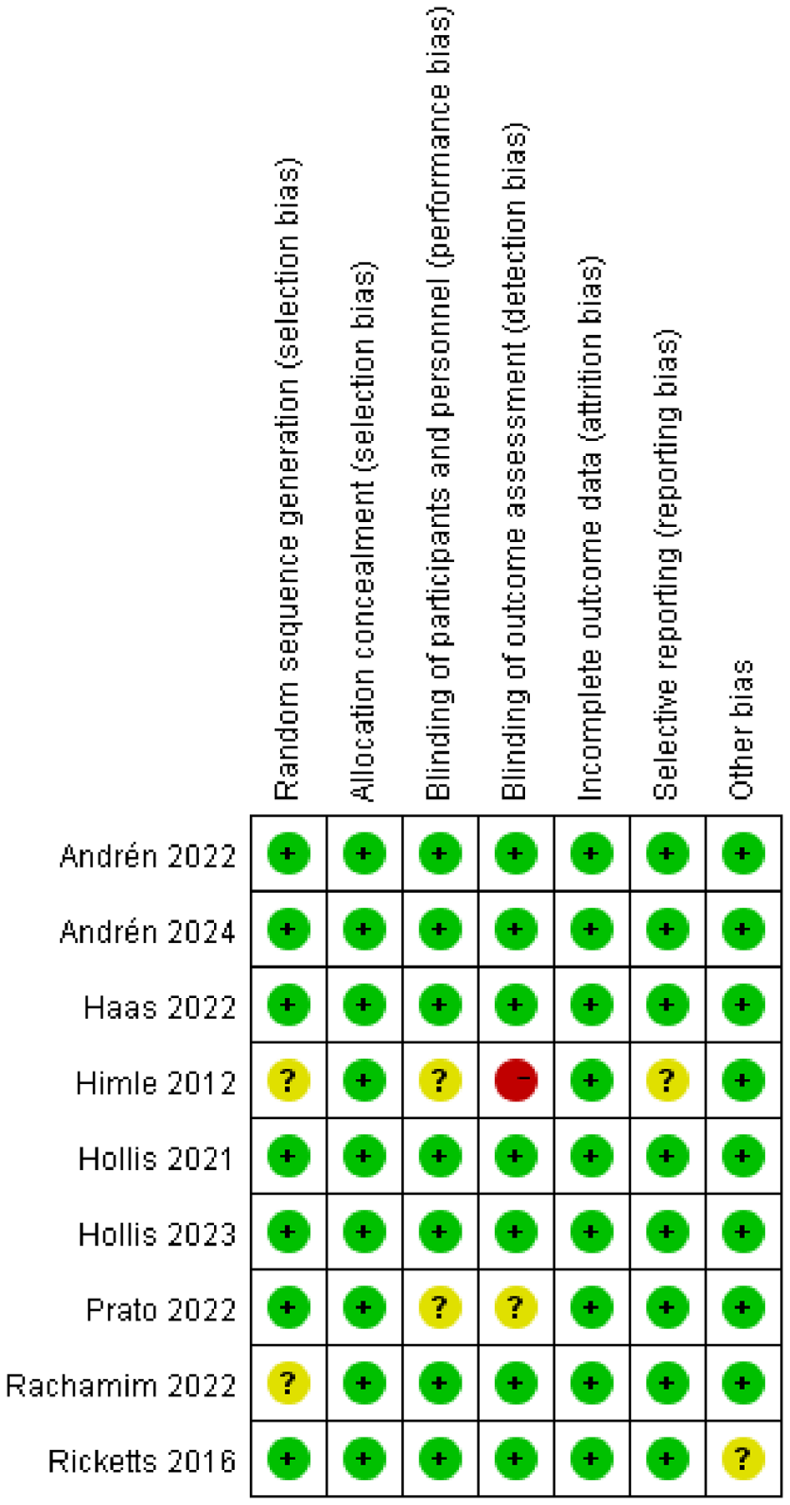
The risk-of-bias assessment of the studies.

Details of the risk-of-bias assessment of the studies are reported in **Figure 2**. Visual inspection of the plot and Egger’s test suggested that no publication bias was observed, with the funnel plot showing a relatively symmetrical distribution (Egger’s test, *P*>0.05). The funnel plot and Egger’s test of studies was presented in **Supplementary S3**.

### 3.3 Efficacy outcomes

#### 3.3.1 Efficacy of TSORBT on YGTSS-TTSS

**Figure 3:**
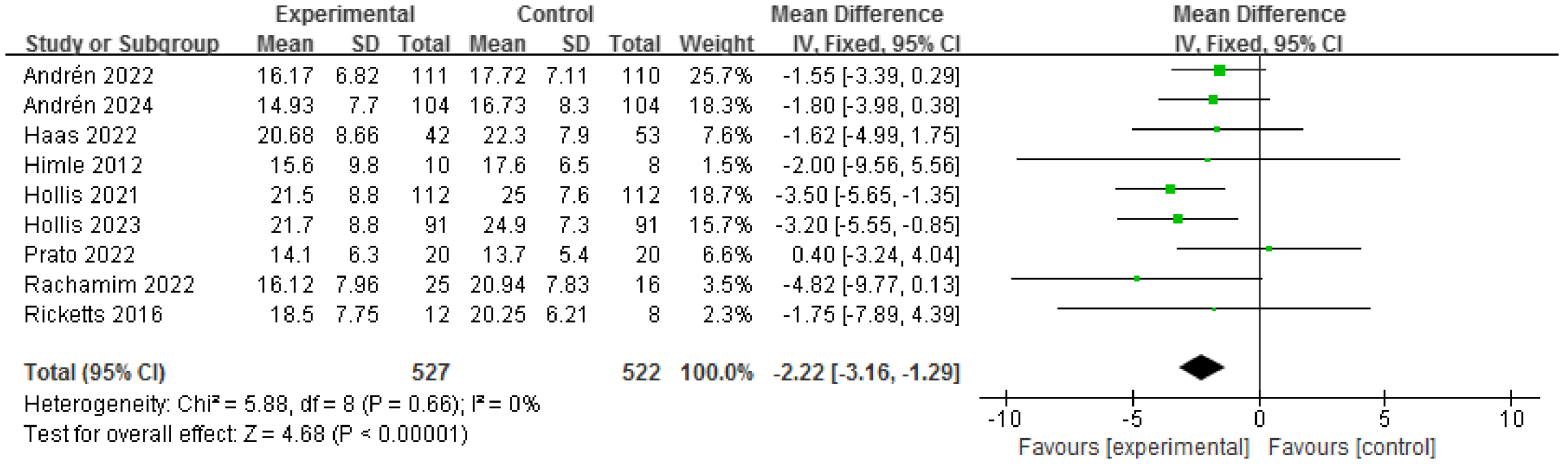
Forest plot for efficacy of TSORBT on YGTSS-TTSS.

Based on the meta-analysis, the TSORBT had significantly more efficacy on YGTSS-TTSS compared with control group (MD = -2.22, 95% CI: [-3.16, -1.29], *P*<0.00001; I^2^ = 0%), which is considered a conclusive superiority (**Figure 3**). Due to age factors may affect the results, when removing the studies by Haas et al., which the Participants included were adults (age >18 years), the significant efficacy had persisted (MD = -2.27, 95% CI: [-3.24, -1.31], *P*<0.00001; I^2^ = 0%) (**Supplementary S4**).

Long-term follow-up studies of TSORBT for TD have been exceedingly rare. There are two studies to demonstrate the long-term effectiveness (beyond 6 months) of ERP. The analysis revealed that the TSORBT intervention reduced YGTSS-TTSS after 12-month follow-up with an effect size of (MD= -2.45, 95% CI: [-4.04, -0.85], *P*=0.003; I^2^ = 0%) (**Supplementary S5**). Sensitivity analysis was employed to address this heterogeneity in the YGTSS-TTSS outcome. There is no significant heterogeneity even after removing any of the studies (**Supplementary S6**).

#### 3.3.2 Efficacy of TSORBT on YGTSS-MTS

**Figure 4:**
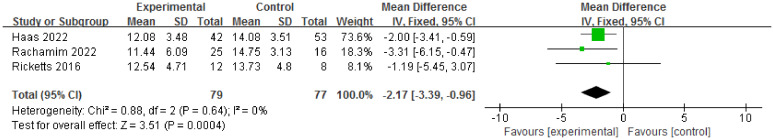
Forest plot for efficacy of TSORBT on YGTSS-MTS.

Three RCTs involving 156 patients evaluated YGTSS-MTS. There was a significant reduction in the motor tic score at the endpoint compared to the control (MD = -2.17, 95% CI: [-3.39, -0.96], *P*=0.0004; I^2^ = 0%) **Figure 4**.

#### 3.3.3 Efficacy of TSORBT on YGTSS-VTS

**Figure 5:**
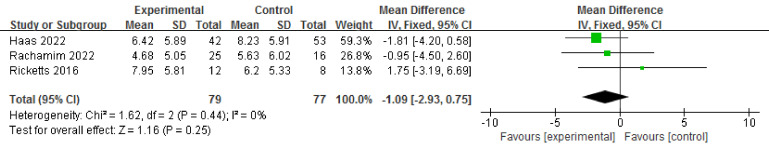
Forest plot for efficacy of TSORBT on YGTSS-VTS.

Three RCTs involving 156 patients evaluated YGTSS- VTS. There was no significant reduction in the vocal tic score at the endpoint compared to the control (MD= -1.09, 95% CI: [-2.93, 0.75], *P*=0.25; I^2^ = 0%) **Figure 5**.

#### 3.3.4 Efficacy of TSORBT on YGTSS-impairment score

**Figure 6:**
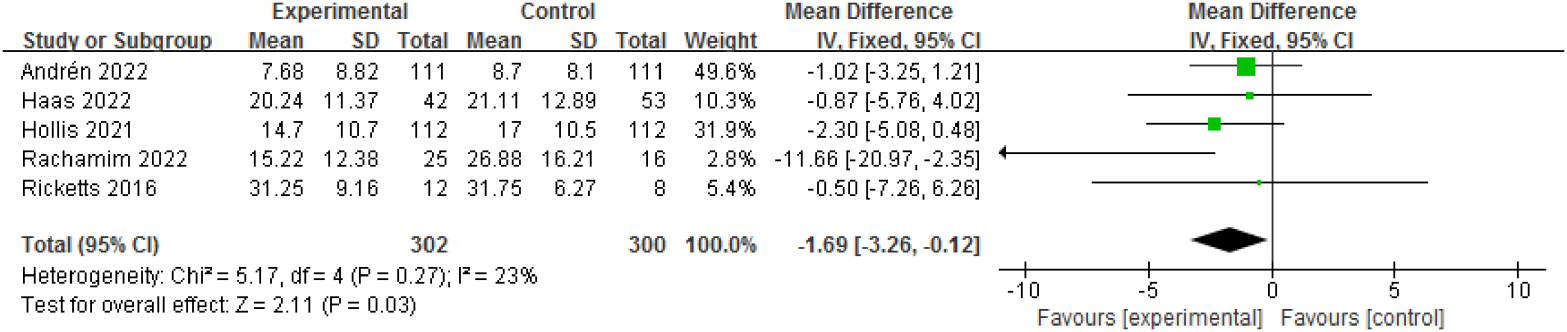
Forest plot for efficacy of TSORBT on YGTSS-impairment score.

Five RCTs involving 602 patients evaluated YGTSS-impairment score. There was a significant reduction in YGTSS-impairment score at the endpoint compared to the control (MD= -1.69, 95% CI: [-3.26, -0.12], *P*=0.03; I^2^ = 23%) **Figure 6**.

#### 3.3.5 Efficacy of TSORBT on PTQ score

**Figure 7:**
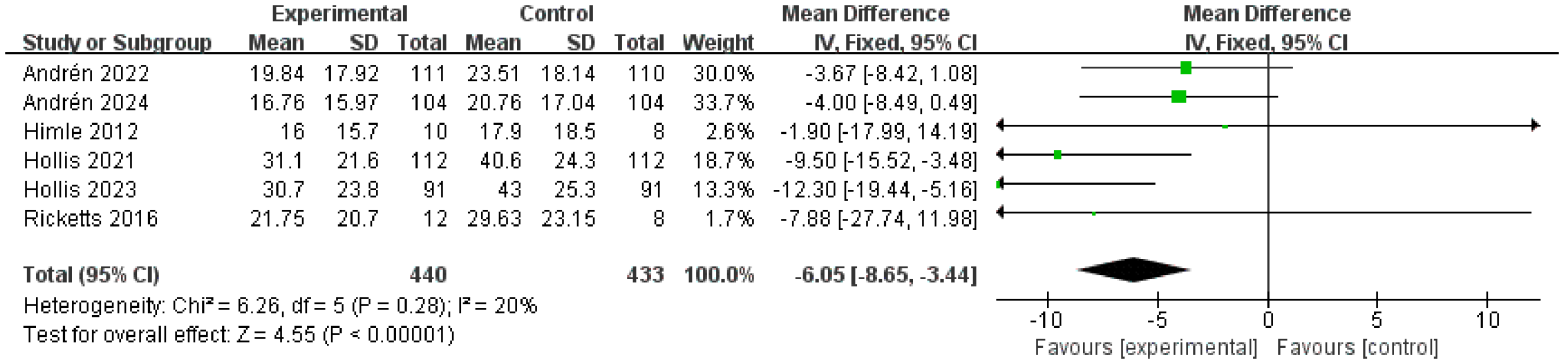
Forest plots for the PTQ score outcome with the TSORBT.

Six RCTs involving 873 patients evaluated PTQ score. There was a significant reduction in PTQ score at the endpoint compared to the control (MD= -6.05, 95% CI: [-8.65, -3.44], *P*<0.00001; I^2^ = 20%) **Figure 7**.

#### 3.3.6 Subgroup analysis

**Figure 8:**
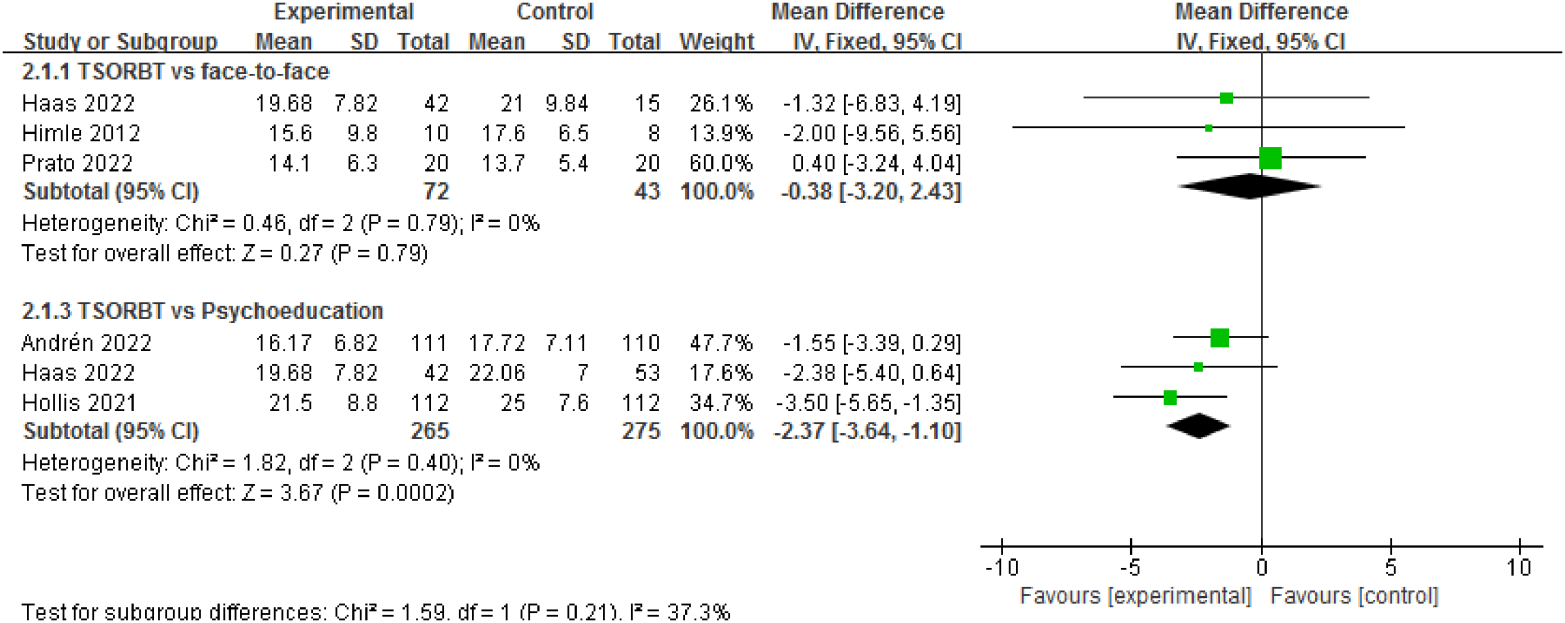
Forest plots of the subgroup analysis by different comparisons for the efficacy of TSORBT.

In a subgroup analysis, the YGTSS-TTSS data related to the f2f and online psychoeducation were included. Three RCTs involving 115 patients evaluated YGTSS-TTSS between TSORBT and f2f. There was no significant difference observed in the reduction of the YGTSS-TTSS at the endpoint (MD= -0.38, 95% CI: [-3.20, 2.43], *P*=0.79; I^2^ = 0%). Three RCTs involving 540 patients evaluated YGTSS-TTSS between TSORBT and online psychoeducation. There was a significant difference observed in the reduction of the YGTSS-TTSS at the endpoint (MD= -2.37, 95% CI: [-3.64, -1.10], *P*=0.0002; I^2^ = 0%) **Figure 8**.

Furthermore, we evaluated all the results using the GRADE pro (https://gdt.gradepro.org/app). The GRADE quality of the supporting evidence for the outcome was high (**Supplementary S7**).

## 4 Discussion

In this systematic review and meta-analysis, we managed a comprehensive analysis to identify the efficacy of TSORBT for TD. We focus on tic severity and PTQ. We analyzed data from nine studies involving 1049 patients with TD, and found significant reductions in YGTSS-TTSS, YGTSS-MTS, YGTSS-impairment score and PTQ. However, our findings did not significantly impact vocal tic measured by the YGTSS-VTS. Furthermore, subgroup analysis found that, in contrast to studies using f2f BT, TSORBT shows almost the same effect. However, when compare with online psychoeducation, there was a significant difference observed in the reduction of the YGTSS-TTSS at the endpoint. To our knowledge, this is the first systematic review and meta-analysis of TSORBT for TD.

TD are commonly encountered in pediatric neurology clinics. Individuals with tic disorders, including TS, face numerous psychological and social difficulties due to persistent tics, such as peer stigmatization and diminished quality of life, which can also affect their families (34, 35). The limited availability of trained therapists in numerous countries hinders access to behavioral treatments for tic disorders (36, 37), resulting in a mere one-fifth of young individuals with TD being able to receive therapy (10). The integration of information technology and treatment has led to the development of a therapy that involves real-time communication between patients and therapists through Internet (23, 32). TSORBT provides a standardized treatment platform and a professional treatment manual, both designed to enhance participants’ effectiveness and efficiency. This intervention does not require therapists to have graduate-level education or prior experience in treating tic disorders. Instead, they receive training on the platform and its contents, along with regular expert supervision. Additionally, the platform integrates supplementary information and instructions specifically for parents/caregivers, enabling them to optimally support their children/adolescents. The TSORBT improves treatment adherence and reduces the time commitment for both therapists and patients (38), it also reduces the cost for patients (39), allowing them to receive treatment without disrupting their school or work commitments (22, 40). Moreover, this intervention presents a potential solution to mitigate the stigma associated with in-person therapy visits and also had a positive impact on helping users,mental health (10).

The first report about the efficacy of BT delivered via telehealth dates to the study by Himle et al. (29) at 2012. Previous studies included small sample sizes and had short follow-up periods (1, 22, 29, 30), which didn’t had enough evidence to prove its advantage. Recently, three RCTs exceeded more 500 participants included (24, 31, 32), have further explored the effects of TSORBT on TD and added long-term follow-up data. In the trial of Haas et al. (24), they investigated treatment effects of iCBIT on tics in adult patients with TD compared to placebo and f2f CBIT and found that iCBIT is superior to placebo and not inferior to f2f in the reduction of tics. In the study of Hollis et al. (31), they evaluate the effectiveness of internet-delivered, therapist-supported, and parent-assisted ERP for treatment of tics in children and young people with TS or chronic tic disorder in England. This is the first adequately powered, randomized, controlled trial assessing online delivery of therapist-supported ERP for tics. They found that the acceptability and safety of the intervention were high. Importantly, the therapeutic effect was durable and even increased slightly at 6 months. Interestingly, only recently, Andrén et al. (32)reported both internet-delivered ERP or education were associated with clinically meaningful improvements in tic severity, but ERP led to higher response rates at little additional cost.

In this study, our meta-analysis indicates that the TSORBT had significantly more efficacy on YGTSS-TTSS compared with other interventions after end of treatment over 2 to 6 month follow-up period. Long-term follow-up is particularly important in the evaluation of treatments for TD, as tics naturally wax and wane over time. Two RCTs reports on 12-month follow-up to establish the long-term efficacy, the results showed that TSORBT is an effective intervention with durable benefits extending for up to 12 months, even 18 months (only in Hollis et al. (23)). This effect is also reflected in YGTSS-MTS, YGTSS-impairment score. Nevertheless, the YGTSS-impairment score had a wide confidence interval, especially the lower limit to -3.26, while the upper limit is close to 0, indicates that there may be some uncertainty in the results. Although statistically significant, clinical significance needs to be considered, suggesting that the results need to be interpreted with caution.

In addition, our observations revealed no significant improvement in vocal tics. This finding aligns with Rachamim et al.’s report (22), which documented minimal improvement during active intervention but marked amelioration during follow-up. Similarly, Hans et al. (24) observed a progressive reduction in vocal tic scores after six-month follow-up, though this change lacked statistical significance (*P*=0.06). These gradual improvements may reflect continued practice and supplemental sessions during follow-up. Yet, the delayed efficacy of the intervention on vocal tics, in contrast to motor tics, could potentially stem from distinct neurobiological mechanisms and the heightened susceptibility of vocal tics to environmental influences (41). At the same time, only three studies followed vocal tics, adding to the heterogeneity of the results. This suggests that future studies need to pay more attention to the effects of BT on vocal tics, and the follow-up time should be extended.

Subgroup analysis revealed comparable efficacy between TSORBT and f2f BT. However, TSORBT demonstrated significantly greater reduction in YGTSS-TTSS scores compared to online psychoeducation. TSORBT provides a standardized treatment platform and a professional treatment manual, both designed to enhance participants’ effectiveness and efficiency. The platform incorporates additional guidance specifically for parents, enabling them to provide optimal support. Furthermore, built-in progress tracking and regular therapist reminders significantly improve treatment adherence. Therapists not only guided participants in platform navigation but also provided consistent reminders and positive reinforcement for module completion and therapeutic progress. In contrast, the online psychoeducation control condition focused on tic disorder epidemiology (history, prevalence, risk factors) and general health recommendations, without specific tic-management strategies.

Particularly, when compared with traditional face-to-face therapy, TSORBT offers distinct advantages by enabling flexible participation timing, thus minimizing school/work absenteeism and eliminating the need for long-distance travel to specialized centers. These accessibility benefits contributed to a two-fold greater likelihood of positive treatment response among participants. A key distinction between online delivery and face-to-face behavioral therapy for tics lies in three aspects: reduced therapist time requirements, lower necessary skill level for therapists, and decreased costs. The total therapist contact time was approximately 2.5 hours, compared to 9–10 hours in comparable evidence-based face-to-face behavioral therapy for tics (31). Given the shortage of therapists specialized in tic disorders and limited access to behavioral therapy, TSORBT has significant potential to expand the availability of effective behavioral interventions.

Based on our current findings, we propose three key directions for future research: First, studies should examine the durability of TSORBT treatment effects through extended follow-up periods. Second, research should explore potential synergies between TSORBT and complementary digital health technologies (e.g., smartphone-based monitoring applications) to improve both treatment accessibility and adherence. Third, a stepped-care model could be developed where TSORBT serves as a first-line behavioral intervention for tic disorder screening and initial treatment. In this model, non-responders to the digital intervention could transition to either blended online-offline approaches for complex cases or other intervention (e.g. deep brain stimulation) (42, 43), thereby optimizing clinical resource allocation while expanding treatment access for individuals with transient or chronic tic disorders. On the whole, the ultimate purpose of our study on TSORBT for tics is not to replace ftf therapy, but to allow this scarce resource to be better targeted to those who need it most and to provide an effective intervention for those with limited access to BT. This meta-analysis demonstrates that TSORBT represents a promising treatment alternative for TD patients.

Our study has several limitations that should be acknowledged. Firstly, the sample size of some studies was relatively small, which may have limited the statistical power of our findings. We also found that in some studies, the dropout rate increased over time, especially in the face-to-face group, which could have affected the results. Although most outcomes demonstrated low heterogeneity, this finding should be interpreted cautiously as only nine studies were included. Furthermore, other heterogeneity may exist due to variations in participants’ age ranges and comorbid conditions across studies. Secondly, the wide scope of our experiment, involving multiple countries, introduces potential variations in conditions that could impact the treatment effect. In addition, there are some differences in online behavioral interventions. For example, in the studies of Himle et al. (29) and Ricketts et al. (30), CBIT was delivered by a therapist via video instead of f2f. In the study of Andrén et al. (32), Therapist-Supported Internet-Delivered ERP was accompanied by both therapists and parents, and in the study of Rachamim et al. (22), a therapist not only guided participants in the use of the online platform, but also repeatedly reminded and praised them to complete relevant modules and for making progress. Consequently, further targeted studies are necessary to account for these differences. Thirdly, the short duration of treatment in the identified studies, typically ranging from 2 to 3 months, may restrict the comprehensive understanding of outcomes. Lastly, despite our thorough search, the possibility remains that unpublished studies have not been identified, which could potentially introduce bias to our findings. Although Visual inspection of the plot and Egger’s test suggested that no publication bias was observed, we should indeed be interpreted with caution due to potential error.

## 5 Conclusions

TSORBT represents an efficient public mental health intervention that enhances access to BT for tics, demonstrating efficacy comparable to face-to-face BT while outperforming online psychoeducation. To further validate these findings, rigorously designed studies, particularly those evaluating long-term treatment durability, are warranted to consolidate the evidence base.
